# High permittivity processed SrTiO_3_ for metamaterials applications at terahertz frequencies

**DOI:** 10.1038/s41598-018-33251-y

**Published:** 2018-10-15

**Authors:** Cyrielle Dupas, Sophie Guillemet-Fritsch, Pierre-Marie Geffroy, Thierry Chartier, Matthieu Baillergeau, Juliette Mangeney, Jean-François Roux, Jean-Pierre Ganne, Simon Marcellin, Aloyse Degiron, Éric Akmansoy

**Affiliations:** 1 Univ Limoges, CNRS, SPCTS UMR 7315, Ctr Europeen Ceram, F-87068 Limoges, France; 2 CIRIMAT, Université de Toulouse, CNRS, INP, UPS, F-31062 Toulouse, France; 3 Univ Paris 06, Univ D. Diderot, CNRS, Ecole Normale Super, Lab Pierre Aigrain, UMR 8551, F-75231 Paris 05, France; 4 IMEP - LaHC UMR 5130, Université Savoie Mont-Blanc, F73376 Le Bourget du Lac, France; 5 Thales Research & Technology, Route Départementale 128, 91767 Palaiseau Cedex, France; 6 Institut d’Électronique Fondamentale, Univ. Paris-Sud, Université Paris-Saclay, Orsay, F-91405; UMR8622, CNRS, Orsay, F 91405 France

## Abstract

High permittivity SrTiO_3_ for the realization of all-dielectric metamaterials operating at terahertz frequencies was fabricated. A comparison of different processing methods demonstrates that Spark Plasma Sintering is the most effective sintering process to yield high density ceramic with high permittivity. We compare this sintering process with two other processes. The fabricated samples are characterized in the low frequency and in the terahertz frequency ranges. Their relative permittivities are compared with that of a reference SrTiO_3_ single crystal. The permittivity of the sample fabricated by Spark Plasma Sintering is as high as that of the single crystal. The role of the signal-to-noise ratio in the measurements at terahertz frequency is detailed.

## Introduction

All-Dielectric Metamaterials (ADM) are the promising alternative to Metallic Metamaterials (MM). Metamaterials give rise to unnatural phenomena such as negative index, sub-wavelength focusing and cloaking. They are engineered materials whose unit cell generally comprises two sub-wavelength building blocks. Their electromagnetic properties are predominantly defined by the geometry of their unit cells. MMs were first demonstrated in the microwave regime. However, going up to the terahertz and the optical domains has been difficult due to ohmic losses and complicated geometries. ADMs rely on the first two modes of Mie resonances of High Permittivity Resonators (HPR)^1–4^. They do not suffer from ohmic losses and consequently benefit of low energy dissipation^2^; moreover, their unit cell is of simple geometry. HPRs at a few tens of microns scale are required for ADMs applications in the Terahertz (THz) range^5^ (see also the design of an ADM-based GRin INdex (GRIN) lens operating in the THz range^6^ and about the role of the mode coupling to ensure negative index at THz frequency^7^).

THz radiation is widely defined as the electromagnetic radiation in the frequency range 0.3–10 THz. It allows to obtain physical data that are not accessible by the means of X-rays or infrared radiation. In this respect, THz radiation offers many applications in imaging, spectroscopy, chemical sensing, astronomy, security, etc. On their part, metamaterials have evolved towards the implementation of optical components^8^. ADMs permit to achieve a great number of fascinating phenomena, e.g., optical cloaking^9^, perfect reflectors^10^, zero-index metamaterials^11^ (see^12^ for a review).

They nevertheless require efficient fabrication processes to develop, namely, processes which lead to high permittivity ceramics that could be structured at the micron scale. The growth of single crystals is a very long process that provides small-sized ceramic. Moreover, after the growth, the fabrication of dielectric metamaterials from the ceramic requires laser micro-machining, which limits the etching depth and the verticality of the walls^5^. In this manuscript, we show that SPS, which is a rather simple and fast fabrication process, makes it possible to fabricate dense high permittivity SrTiO_3_ ceramic suitable for applications at terahertz frequencies. We compare this sintering process with two other methods: tape casting and uniaxial pressing with subsequent conventional sintering. The structural properties of the fabricated samples were investigated by X-ray diffraction (XRD) and Scanning Electron Microscope (SEM). Then, the samples were characterized in the low frequency range and at THz frequencies by the means of Time Domain Spectroscopy (THz-TDS), and their dielectric constant was compared with that of a reference SrTiO_3_ single crystal; namely, we compare the properties of our polycrystalline samples with that of a single crystal. The dielectric constant of the polycrystalline samples fabricated by SPS is as high as that of the single crystal. SPS is the efficient process to manufacture high performance polycrystalline materials. This manuscript thus gathers multidisciplinary results, from chemistry and material science to THz-TDS.

The premise for our study is the demonstration of ADMs in the THz range, and specially metadevices, i.e., efficient functional devices integrating the fascinating properties of metamaterials. The latter can be engineered for real-life applications. In addition, low THz range is an atmospheric transmission window available for large band telecommunications and also for imaging. As the long growth process of single crystals hampers industrial fabrication, we looked for the effective process of fabrication that yields high permittivity SrTiO_3_ ceramics to develop ADMs.

## Results and Discussion

### Structural characterization of the ceramics

**Figure 1:**
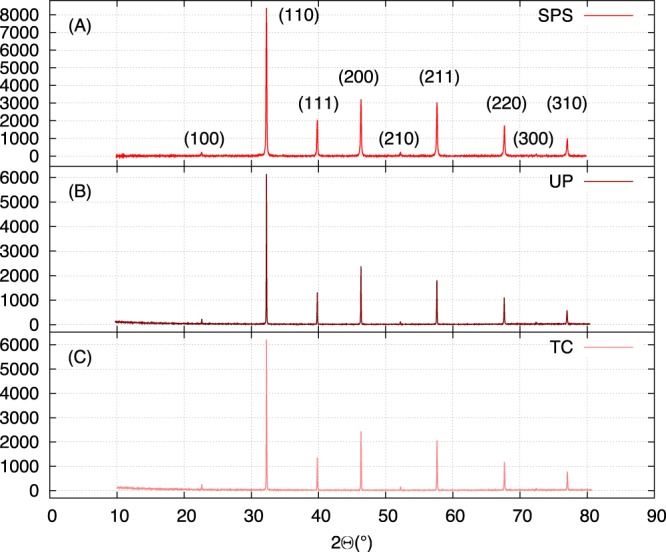
XRD patterns of sintered SrTiO_3_ samples: (**A**) SPS sample, (**B**) UP sample, (**C**) TC sample. The samples are in the same crystalline phase (JCPDS 01-070-8508) SPS stands for Spark Plasma Sintering, UP stands for Uniaxial pressing, TC stands for Tape casting.

**Figure 2:**
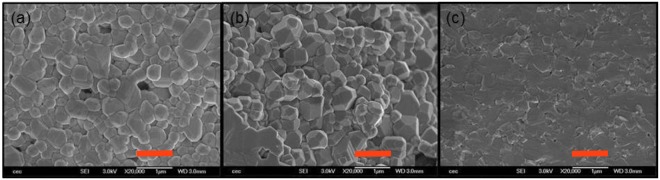
SEM images of sintered SrTiO_3_ samples: (**a**) TC sample, (**b**) UP sample, (**c**) SPS sample. Scale = 1 *μ*m (orange dash). Porosity can be noticed in the TC sample.

XRD analyses were performed on the batches of sintered ceramics and the corresponding images are presented in Fig. 1. Whatever the shaping process, all the samples are in the same crystalline phase, namely, the cubic perovskite structure of SrTiO_3_ (JCPDS 01-070-8508). Then, the relative density *d* of the samples was measured by Archimed’s method (see Table 1)^13^. The relative density of the Tape-Casting (TC) samples is low: *d* ≈ 70%, whereas that of the Uniaxial pressing (UP) samples is improved, ranging from *d* = 90% to 95%. Last, the Spark-Plasma-Sintering (SPS) samples exhibit the highest density: *d* ≥ 99%. In addition, the samples were observed by SEM, from which it can be deduced that, whatever the shaping process, the mean grain size of the samples is about 0.5 *μ*m (see Fig. 2). Consequently, as the chemical composition, the structure and the grain size are identical whatever the implemented process, only the density of the samples can affect their dielectric constant.

**Table 1:** Density and dielectric constant at 1 kHz of SrTiO_3_ samples in dependence of the shaping process.

Shaping process	Sample	Density d	${\varepsilon }_{r}^{^{\prime} }$/tan δ (1 kHz)
Tape casting & conventional sintering	TC-STO-6a TC-STO-PM1 TC-STO-1	67% 71% 73%	236/0.022 253/0.02 —
Uniaxial pressing & conventional sintering	UP-STO-2 UP-STO-4 UP-STO-7	91% 93% 95%	280/0.02 280/0.015 289/0.002
Spark Plasma Sintering	SPS-035 SPS-036 SPS-100 SPS-101	≥99% ≥99% ≥99% ≥99%	338/0.002 360/0.005 327/0.002 —

SPS stands for Spark Plasma Sintering, UP stands for Uniaxial pressing, TC stands for Tape casting.

### Dielectric characterization of the ceramics

#### Dielectric constant at 1 kHz

The dielectric constants were first compared at 1 kHz and the results are given in Table 1 in dependence of the density. The relative permittivity of the TC samples slightly increases with the density *d* from ${\varepsilon ^{\prime} }_{r}$ = 236 to 253, while the dielectric losses remain around tan *δ* = 0.02. The relative permittivity of the UP samples increases from ${\varepsilon }_{r}^{^{\prime} }$ = 280 to 289, as the density increases from *d* = 90% to 95%, while the dielectric losses significantly decrease from tan *δ* = 0.02 to 0.002. Last, the SPS samples exhibit the highest relative permittivity: ${\varepsilon }_{r}^{^{\prime} }$ > 320. This process therefore yields very dense samples (*d* ≥ 99%), whose relative permittivity at 1 kHz is the highest (${\varepsilon }_{r}^{^{\prime} }$ > 320) of the three batches and whose dielectric losses are very low (tan *δ* ≤ 0.005).

#### Dielectric constant at terahertz frequencies and signal-to-noise ratio

**Figure 3:**
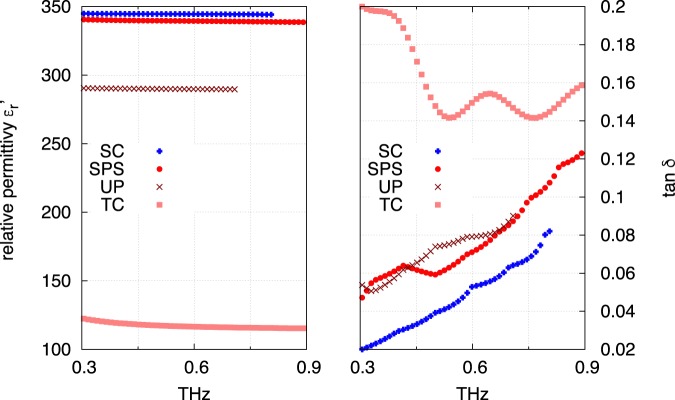
Permittivity ${\varepsilon }_{r}^{^{\prime} }$ and dielectric losses tan *δ* in the terahertz range measured by THz-TDS of different processed SrTiO_3_ samples.

**Figure 4:**
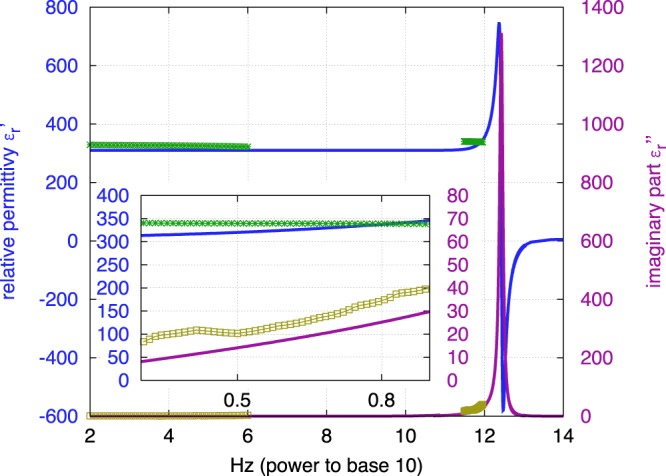
PH model of the dielectric response of SrTiO_3_ between 10^2^ and 10^14^ Hz: *ε*_*r*_ (*ω*) = ${\varepsilon }_{r}^{^{\prime} }$ + *i*${\varepsilon }_{r}^{^{\prime \prime} }$ (${\varepsilon }_{r}^{^{\prime \prime} }$ = tan *δ* × ${\varepsilon }_{r}^{^{\prime} }$) and measurements in the low frequency (100 Hz–1 MHz) and the THz frequency ranges of SPS sample (green and yellow-green points). Inset: zoom between 0.3 and 0.9 THz.

Furthermore, we performed the dielectric characterization in the terahertz range, which was carried out by measuring the transmission of the samples. A SrTiO_3_ single crystal (Verneuil growth and cubic perovskite structure (CrysTec GmbH http://www.crystec.de/)) serves as a reference for physical parameters. Its dielectric constant was measured in the 0.2–0.9 THz range: the relative permittivity is ${\varepsilon }_{r}^{^{\prime} }\simeq 345$, while the dielectric losses practically linearly increase from tan *δ* = 0.02 to 0.08 (Fig. 3). These results are in good agreement with those of ref.^14^. This behavior is also in good agreement with the classical pseudoharmonic (PH) model (see below and Fig. 4)^15,16^. The dielectric constant of three differently processed samples are given in Fig. 3 as well. The TC samples show low relative permittivity (${\varepsilon }_{r}\simeq 117$) and high losses (tan *δ* > 0.14). Due to the porosity, the measured sample is a composite medium made of SrTiO_3_ and air, and its dielectric constant is an effective quantity *ε*_*eff*_ which is commonly described by the Bruggeman effective medium approximation (EMA)^15^. Porosity consequently lowers the relative permittivity ${\varepsilon }_{r}^{^{\prime} }$ and increases the losses tan *δ*. The UP sample exhibits improved relative permittivity (${\varepsilon }_{r}^{^{\prime} }\simeq 290$) and losses which also increase with the frequency in accordance with the PH model. The higher density similarly leads to increased relative permittivity ${\varepsilon }_{r}^{^{\prime} }$ in the terahertz range. Indeed, the relative permittivity of the SPS sample is actually close to that of the single crystal, i.e. ${\varepsilon }_{r}^{^{\prime} }\simeq 340$, while the dielectric losses, increasing from $\tan \,\delta \simeq 0.055$ at 0.3 THz to $\simeq 0.012$ at 0.9 THz, are about 1.3 times those of the single crystal in this frequency range. We ascribe this point to the coexistence of Ti^4+^ and Ti^3+^ cations in the sample (A longer annealing after sintering could have lowered the numbers of Ti^3+^ cations.) and to grain boundaries which do not exist in single crystals. Defects and grain boundaries may increase the mobility of atomic species and the losses. Figure 3 clearly demonstrates that the higher the density, the higher the relative permittivity ${\varepsilon }_{r}^{^{\prime} }$ and the lower the dielectric losses tan *δ*.

**Figure 5:**
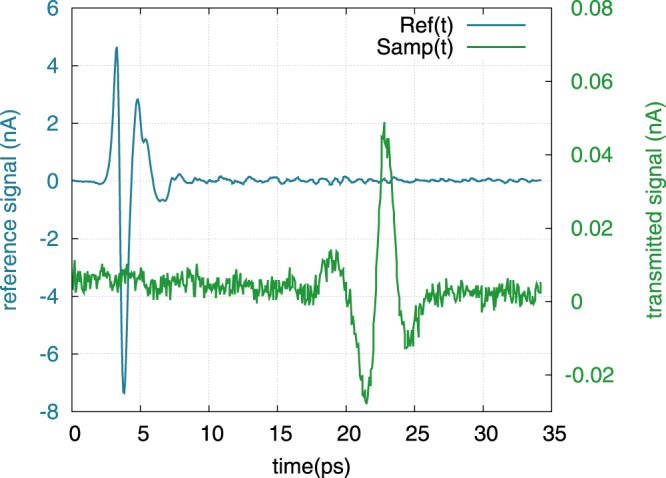
Measurement of the THz time domain signals: direct reference signal *Ref*(*t*) without sample (blue, left axis) and signal transmitted by sample SPS-036 *Samp*(*t*) (green, right axis).

**Figure 6:**
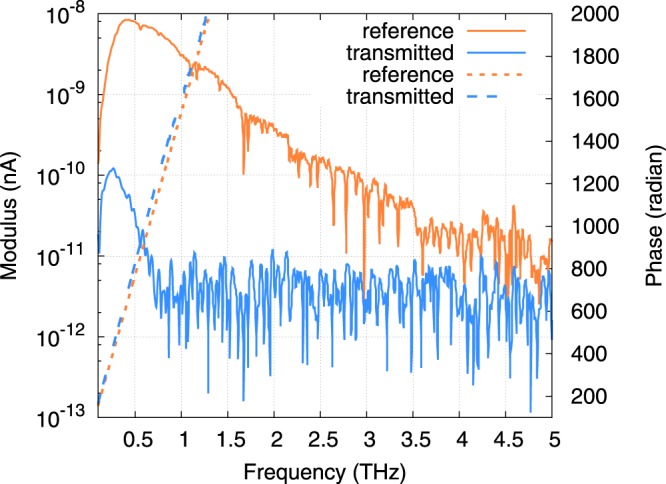
Fourier transformed spectra: modulus (continuous lines) and phase (dashed lines) of the reference (orange lines) and transmitted (blue lines) signals for the sample SPS-036. The modulus of the reference signal peaks around 400 GHz at a maximum of 8.5 nA, the noise floor at high frequency is estimated to be around 8 pA. The deep narrow lines at 1.1, 1.7, 2.62 THz, etc. are the signature of residual water vapor absorption in the chamber of measurement. The phase of both signals appear to vary almost linearly with the frequency.

**Figure 7:**
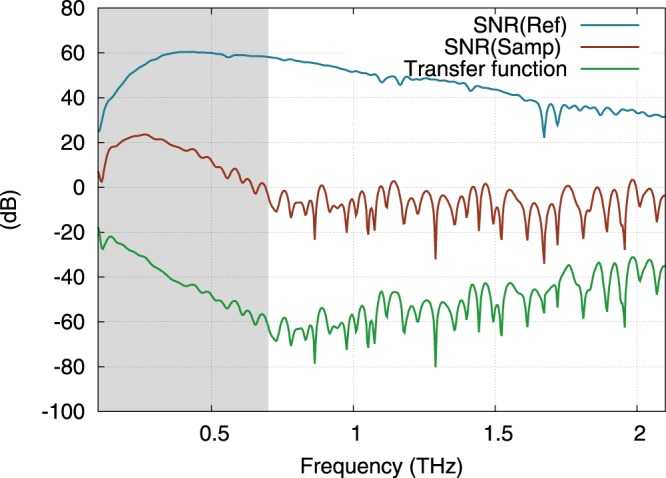
Signal-to-Noise Ratios: SNR (Ref) of the experimental setup (blue) defined as the modulus of the reference signal minus the shot noise level of 8pA (see Fig. 6), modulus of the Transfer function (TF) of the sample (green) and SNR of the transmitted signal (brown) defined as SNR (Samp) = SNR (Ref) + Transfer function (in dB). The range of reliability for the determination of the sample optical properties is fixed by the condition SNR (Samp) ≥ 0 dB. It ranges from 0.2 to 0.7 THz in this case (shaded area).

To perform the THz-TDS measurements, the temporal shape of the THz signal transmitted by the sample *Samp*(*t*) and its delay are compared with a reference measurement *Ref*(*t*), made without the sample. Spectra of the both signals are then calculated. Figure 5 shows some typical time domain signals recorded with one of the two THz TDS setups. The reference pulse *Ref*(*t*), peaking at 3.8 ps, shows a classical bipolar shape with an approximate pulsewidth of 5 ps. It is followed by small oscillations due to residual absorption of the present water vapor in the chamber of measurement where the relative humidity is maintained under 10% at 23 °C. The signal *Samp*(*t*) transmitted by the sample SPS-036 (thickness 334 *μ*m) consists in a THz pulse that is delayed by 19 ps as compared to the main pulse of *Ref*(*t*). From this time of flight, we estimate that the group refractive index of the sample is about *n*_*g*_ = 18. This very high index leads to strong reflection of the THz signal at the sample interfaces so that only 20% of the incident field is transmitted by the interfaces of the 334 *μ*m thick parallel plate. These reflection losses add to the absorption losses of the sample. Consequently, the signal amplitude is reduced by a factor of 180 corresponding to total losses of −45 dB. However, more insight about the sample properties is obtained from the numerically calculated spectra of *Ref*(*t*) and *Samp*(*t*) signals plotted in Fig. 6. The modulus of the spectrum *Ref*(*t*) peaks at 8.5 nA around 400 GHz and is linearly decreasing (in dB) until it reaches the white noise floor around 4.5 THz. Besides, the modulus of the spectrum *Samp*(*t*) decreases till it reaches the noise level around 0.7 THz, which demonstrates that absorption losses of the sample increase with the frequency. This is consistent with the PH model (see Fig. 4). Also note that in the range 0.2–0.7 THz, the phase of the signal *Samp*(*t*) linearly varies exhibiting no phase shift that would be the signature of an absorption peak, thus confirming the continuous evolution of the modulus. From these two spectra (modulus and phase), we calculate the transfer function of the sample shown in Fig. 7. Before retrieving the dielectric constant of the sample from this transmission, we should determine the limit of our experimental procedure. Actually, the ability of such an experimental setup to yield reliable results over a frequency range strongly depends on two points. On the one hand, it depends on the frequency dependent performances of the setup i.e., the Signal to Noise Ratio (SNR)^17^ and on the other, on the absorption losses introduced by the sample^18,19^. The limit of reliable exploitation of the data is obtained when the SNR of the signal transmitted (Sampl) by the sample reaches unity. Usually, in such a THz-TDS experiment, the main noise contribution comes from the relative intensity noise (RIN) of the laser that induces fluctuations of the THz emitted power. However, it has been shown that for samples with low transmission (i.e. low level of THz signal impinging the detector) the shot noise of the detector is the major contribution^17^. Here, this corresponds to the constant level of noise that is estimated to be around 8 pA (see Fig. 6). Therefore, as the sample strongly attenuates the transmitted signal, we estimate that the upper frequency limit for reliable data analysis is 0.7 THz for this sample (see Fig. 7). On the other hand, we estimate the lower frequency limit of reliability to be around 200 GHz. Below this frequency, the data is inaccurate due to the rapid drop of the signals to a very low level, while the frequency resolution of the experiment is low (typ. 4 GHz). The numerical comparison of both the reference and sample spectra is consequently very fluctuating at low frequency.

### Measurements *vs* theoretical model

The dielectric response *ε*_*r*_ (*ω*) of SrTiO_3_ is dispersive, because of the lattice vibrations, namely, the optical phonons^16,20^. Their frequency is in the THz range, and we are concerned by the Transverse Optical phonon of lowest frequency (TO1). The TO1 phonon frequency of SrTiO_3_ is 2.70 THz^16,20^. The dielectric function *ε*_*r*_ (*ω*) is described by the PH model (see eq. 2 in ref.^15^), which is reported in Fig. 4 or by the the four-parameters semi-quantum (FPSQ) model^14,20^. The two models are equivalent at the operating frequency. Losses (imaginary part of *ε*_*r*_ (*ω*) = ${\varepsilon }_{r}^{^{\prime \prime} }$) resulting from the TO1 phonon greatly increase at terahertz frequencies. Measurements in both frequency ranges are also reported in Fig. 4 and are in good agreement with the PH model.

## Conclusions

Fully dense SrTiO_3_ material sintered by SPS, with a permittivity as high as that of the single crystal, is suitable for ADM applications at THz frequencies. The dielectric constant is in good agreement with the PH model. SPS is the effective process required by ADMs to develop.

## Methods

### Fabrication of the ceramic samples

Commercial SrTiO_3_ powder (Marion Technologies http://www.mariontechnologies.com/nanomateriaux/), with a mean grain size of 0.5 *μ*m, is used to shape ceramic according to three different processes: Tape Casting (TC), Uniaxial Pressing (UP) and Spark Plasmas Sintering (SPS), the two former involving conventional sintering. Each fabrication process was implemented to prepare a batch of a few samples under similar conditions from the same SrTiO_3_ powder. Their chemical composition is consequently the same.

#### Tape casting

Tape casting makes it possible to fabricate thin ceramic sheets with controlled thickness ranging from several tens to a few hundred micrometers^21^. A suspension consisting of the ceramic powder dispersed in a solvent, with the help of a dispersant and containing a binder and a plasticizer, is cast onto a fixed support (Mylar® film). Once the solvent is evaporated, the thickness of the flexible green ceramic tape is around 100 *μ*m. Disks are cut in the tape by a laser beam to avoid stresses in the green tape. These disks are then debinded and pre-sintered at 1100 °C, before undergoing conventional sintering at a temperature between 1320 °C and 1350 °C in air for 1 h. These samples are referred to as TC in the manuscript.

#### Uniaxial pressing

The SrTiO_3_ powder is dispersed in water with addition of a binder, then granulated by spray-drying. A controlled amount of SrTiO_3_ granules is uniaxially pressed into a steel matrix at a pressure of 200 MPa. Obtained green ceramic pellets are then debinded and pre-sintered at 1100 °C, before conventional sintering at 1330 °C. Then, the ceramics pellets are polished until their thickness is a few hundred micrometers. These samples are quoted UP in the manuscript.

#### Spark Plasma Sintering

Spark Plasma Sintering is a sintering process which relies on the heating by a pulsed electric current combined with high uniaxial pressure (around 75 MPa)^22,23^. The SrTiO_3_ powder is set into a carbon graphite die, through which the current is conducted. This process has several advantages because it allows fast heating and the possibility to obtain fully dense samples at comparatively lower sintering temperatures. The grain growth is greatly reduced, while the ceramic samples rapidly get very dense (usually above 98% in a 20 min cycle) (See Supplementary Material for additional data). The sintered SrTiO_3_ ceramics are dark blue colored, which typifies the presence of Ti^3+^ cations^24^. Indeed, during the sintering, the reducing atmosphere educes Ti^4+^ cations into Ti^3+^ cations. Samples are then annealed at 850 °C during a couple of hours in air so as to re-oxidize the Ti^3+^ cations in the sample. These samples are a few hundred micrometers thick and are quoted SPS here.

### Structural and dielectric characterization methods

The crystalline structure and phase purity were first observed at room temperature *via* XRD measurements using a Bruker D4 diffractometer. Then, the grain size and the morphology of the sintered ceramics were observed using SEM. Furthermore, dielectric characterization was carried out in the kHz and the THz ranges in order to determine the dielectric constant (relative permittivity ${\varepsilon }_{r}^{^{\prime} }$ and dielectric losses tan *δ*). On the one hand, the low frequencies dielectric measurements (100 Hz–1 MHz) were performed by the means of an Impedance Analyzer Agilent 4294 A. In the kHz range, the dielectric constant mainly depends on the chemical composition, the structure, the grain size and the density of the ceramics^25^.

On the other hand, the terahertz dielectric properties were measured by the means of two THz-TDS setups that we shortly describe^26,27^. Both setups are based on a Ti:Sa laser (pulse duration 15 or 50 fs, central wavelength 800 nm, 75 MHz repetition rate) whose beam is split into a pump beam and a probe beam (See Supplementary Material). The former is converted into THz radiation (pulse duration in the picosecond range) using GaAs based photoconducting antennaes. The THz beam is spatially shaped using four reflective optics so that it is focused onto both the sample and the detector. The THz pulse transmitted by the sample is measured via electro-optic detection in a ZnTe crystal or via photoconductive sampling. To this aim, it is combined with a delayed laser probe pulse which samples the THz signal. The delay is controlled by a mechanical delay line allowing for time resolution of 1 fs. Using this technique, the temporal shape of the THz signal transmitted by the sample *Samp*(*t*) and its delay with respect to a reference measurement *Ref*(*t*), made without sample, are obtained (see Fig. 5). The data is subsequently Fourier transformed (see Fig. 6) to get the modulus and the phase of the transmission spectrum of the sample. Finally, in the case of dielectric parallel plates such as the samples we are dealing with, the optical index (real and imaginary parts) is retrieved from the experimental transfer function of the sample by inverting the Fabry-Perot equations that describe the THz transmission through the samples^28,29^. More details about THz-TDS technique can be found in^30^.

Besides, before determining the dielectric constant of the samples, we made sure that they could be considered as uniform and homogeneous media that could be characterized through an effective dielectric constant. Thickness variation of each sample was checked to be less than 5% over the characterized surface which was typically 1 cm^2^. The homogeneity of the samples was high enough to assume that scattering effects are low in the considered THz range. Therefore, the determined absorption of the sample was considered to be due to material resonances as explained in our theoretical approach (see Fig. 4).

See Supplementary Material and supporting data for more informations about the fabrication processes (tape casting and SPS), XRD measurements and THz Time Domain Spectroscopy setup.

## Electronic supplementary material

Supplementary Information
