# Supplementary material for: High permittivity processed SrTiO3 for metamaterials applications at terahertz frequencies
Source: Sci Rep. 2018 Oct 15;8:15275. doi: 10.1038/s41598-018-33251-y (PMC6189093; doi:10.1038/s41598-018-33251-y)
Supplement: Supplementary file 1 — Supplementary Information [file 41598_2018_33251_MOESM1_ESM.pdf]

High permittivity processed $SrTiO_3$ for metamaterials applications at terahertz frequencies

Supporting Data & Supplementary Material

Cyrielle Dupas,[1,2] Sophie Guillemet-Fritsch,[2] Pierre-Marie Geffroy,[1] Thierry Chartier,[1] Matthieu Baillergeau,[3] Juliette Mangeney,[3] Jean-François Roux,[4] Jean-Pierre Ganne,[5] Simon Marcellin,[6] Aloyse Degiron,[6] and Éric Akmansoy[6,a)]

[1)] *Univ Limoges, CNRS, SPCTS UMR 7315, Ctr Europeen Ceram, F-87068 Limoges, France*

[2)] *Univ. Paul Sabatier, CNRS, Institut Carnot, CIRIMAT, UMR 5085, F-31062 Toulouse, France*

[3)] *Univ Paris 06, Univ D. Diderot, CNRS, Ecole Normale Super, Lab Pierre Aigrain, UMR 8551, F-75231 Paris 05, France*

[4)] *IMEP - LaHC UMR 5130, Université Savoie Mont-Blanc, - F73376 Le Bourget du Lac Cedex*

[5)] *Thales Research & Technology, Route Départementale 128, 91767 Palaiseau Cedex, France*

[6)] *Institut d'Électronique Fondamentale, Univ. Paris-Sud, Université Paris-Saclay, Orsay, F-91405; UMR8622, CNRS, Orsay, F 91405.*

(Dated: 13 September 2018)

PACS numbers: 78.20.Ci, 77.22.Ch, 81.05.Mh, 78.67.Pt.

Keywords: Dielectrics, Ceramics, Spark Plasma Sintering, THz Time Domain Spectroscopy, Metamaterials

[a)] Electronic mail: eric.akmansoy@u-psud.fr

I. SUPPORTING DATA & SUPPLEMENTARY MATERIAL

A. Tape casting

The suspension consists of the ceramic powder: - 29 %vol, solvent: Ethanol – 42 %vol, dispersant 1 %vol, binder: Methyl methacrylate – 13 %vol and softening agent: Dibutyl phtalate – 15 %vol

B. Spark Plasma Sintering (SPS)

To densify the SrTiO3 nanopowders, SPS was carried out using a Dr. Sinter 2080 device from Sumitomo Coal Mining (Fuji Electronic Industrial, Saitama, Japan). Briefly, 0.5 g of powder was loaded in an 8-mm-inner-diameter graphite die. A sheet of graphitic paper was placed between the punch and the powder as well as between the die and the powder for easy removal of the pellet after sintering. Powders were sintered in vacuum (residual cell pressure $< 10\,Pa$) at 1150°C during 3 minutes, with a pressure of 75 MPa.. An optical pyrometer focused on a small hole at the surface of the die was used to measure and monitor the temperature. The as-sintered pellets presented a thin carbon layer due to graphite contamination from the graphite sheets. This layer was removed by polishing the surface.. Samples appeared dark blue, consistent with the presence of Ti^{3+} caused by the reducing atmosphere used during SPS (low vacuum). 21 SPS pellets were annealed in air atmosphere at 850°C in an attempt to restore the oxygen stoichiometry.

References

E124 International Journal of Applied Ceramic Technology — Voisin, *et al.* Vol. 10, No. S1, 2013

C. X-Ray Diffraction (XRD)

The crystalline structure was investigated by X-ray diffraction analysis using a D4 Endeavor X-ray diffractometer (CuKα = 0.154056 nm and CuKβ = 0.154044 nm, operating voltage 40 kV and current 40 mA). The grain size and morphology of the powders and the microstructure of the sintered ceramics were observed with a scanning electron microscope (SEM, JEOL JSM 6400).

D. Terahertz Time Domain Spectroscopy (THz – TDS)

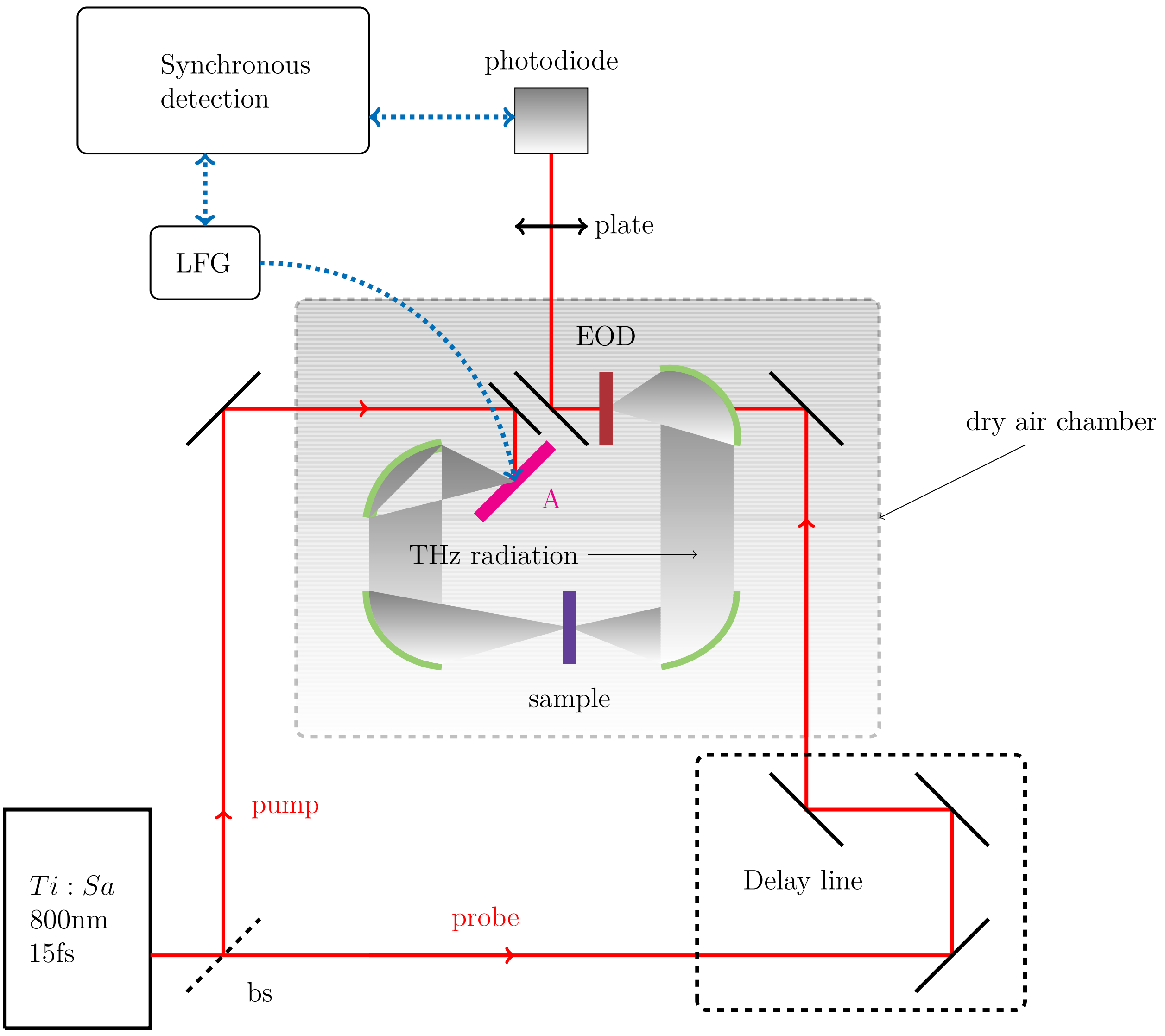

FIG. 1. Schematic layout of the THz-Time Domain Spectroscopy set-up : A stands for photoconductive antenna; EOD for Electro-optic Detection; LFG for Low Frequency Generator; bs for beam-splitter. The shaded area denotes where the THz radiation lies.
